# Supplementary material for: Predicting Malnutrition Risk with Data from Routinely Measured Clinical Biochemical Diagnostic Tests in Free-Living Older Populations
Source: Nutrients. 2021 May 31;13(6):1883. doi: 10.3390/nu13061883 (PMC8226876; doi:10.3390/nu13061883)
Supplement: Supplementary file 1 [file nutrients-13-01883-s001.zip › nutrients-1186648-supplementary.pdf]

Supplementary tables

Supplementary Table S1. NDNS methodology of variables included for analyses with self-computed categories

Variable (unit)	Exact measurement	Computed categories
**Interviewer visit**		
Sex	Ask or record sex of subject	Male, female
Age (years)	Q: What is your date of birth?<br>If day not given, enter 15 for day.<br>If month not given, enter 6 for month.	50 – 59, 60 – 69, ≥ 70
Marital status	Ask or record marital status<br>Q: Are you …<br>Single, that is, never married<br>Married and living with your husband/wife<br>Civil partner in a legally recognised Civil Partnership<br>Married and separated from your husband/wife<br>Divorced<br>Widowed	Single (never married), married or legally recognised civil partnership, divorced or widowed
Qualification gained	Q: Do you have any qualifications from school, college or university, or any qualifications connected with work or from government schemes?<br>If yes, Q: Tell me whether you have any of the qualifications listed. Start at the top of the list and tell me the first one you come to that you passed *(47 answer options)*	Secondary education or less, further education, higher education, other
Region	Record region of interview visit	England: North, England: Central/Midlands, England: South (incl. London), Scotland, Wales, Northern Ireland
Ethnicity	Q: To which of these ethnic groups do you consider you belong? *(15 answer options)*	White British, non-white
Self-assessed general health	Q: How is your health in general? Would you say it was... very good, good, fair, bad, or very bad?	Good, fair, bad
Presence of longstanding illness	Year 1 – 5: Q: Do you have any long-standing illness, disability or infirmity? By long-standing illness I mean anything that has troubled you over a period of time, or that is likely to affect you over a period of time?<br>Year 6 – 9: Q: Do you have any physical or mental health condition(s) or illnesses that have lasted, or are expected to last, for 12 months or more?	Yes, no
Any dietary supplement use in the past year	Check record of taking any supplements in diary<br>Q: Have you taken any vitamins, minerals, fish oil, fibre or other dietary supplements of the type listed on this card in the past year, including prescription and non-prescription supplements?	Yes, no
Appetite	Year 1-5, Q: How would you describe your appetite? Do you have … a good appetite, an average appetite or a poor appetite for someone your age?	Good, average, poor
Any of own teeth	Q: Do you have any of your own, natural, teeth?	Yes, no
Cigarette smoking status	Q: Have you ever smoked cigarettes regularly?<br>Yes, regularly, that is at least one cigarette a day.<br>No, only occasionally<br>Or no, never really smoked cigarettes, just tried them out once or twice.<br>If yes, Q: How long ago did you stop smoking cigarettes regularly?<br>Additional self-completion smoking questionnaire	Never, former, current
Height (cm)	Portable stadiometer	-
Weight (kg)	Weighing scales	-
Body Mass Index (kg/m^2)	Calculated: Weight (kg) / height squared (m^2)	-

**Nurse visit**		
Medication use	Q: Are you taking or using any medicines, pills, syrups, ointments, puffers or injections prescribed for you by a doctor or a nurse? (dietary supplements included as long as prescribed)<br>If yes, Q: Can I see the containers for all prescribed medicines currently being taken?<br>Number of medicines derived.	No medication, 1-4 medicines, 5 or more medicines
**Micronutrient assays**		
Plasma vitamin C (μmol/L)	Based on the Vuileumier and Keck procedure [73], performed on the BMG Labtech FLUOstar OPTIMA plate reader measuring fluorescence.	-
Plasma vitamin B_6 pyridoxal-5-phosphate (PLP) (nmol/L)	Reverse-phase high performance liquid chromatography (HPLC) method with post column derivatisation and fluorimetric detection.	-
Serum vitamin B_{12} (pmol/L)	ADVIA Centaur B_{12} assay using direct chemiluminescence with a releasing agent (sodium hydroxide) and dithiothreitol (DTT) to release the B_{12} from the endogenous binding proteins in the sample.	-
Plasma selenium / zinc (μmol/L)	^{78}Se / ^{68}Zn isotope measurement using an inductively coupled plasma mass spectrometer (IPS-MS) equipped with a dynamic reaction cell (DRC).	-

Supplementary Table S2. Characteristics of study population according to micronutrient deficiency biomarker status (vitamin B_6 PLP, vitamin B_{12}, vitamin C, selenium, zinc) (n = 1518)

Characteristic	No micronutrient deficiencies (n = 789)	1 micronutrient deficiency (n = 443)	2 micronutrient deficiencies (n = 286)	*p* value [1]
Sex, w*omen*	462 (58.6)	250 (56.4)	156 (54.6)	0.467
Age group				<0.001
50–59 years	353 (44.7)	157 (35.4)	66 (23.1)	
60–69 years	267 (33.8)	154 (34.8)	101 (35.3)	
≥70 years	169 (21.4)	132 (29.8)	119 (41.6)	
Ethnic group, *white British*	759 (96.2)	427 (96.4)	281 (98.3)	0.417
Region				<0.001
England: North	153 (19.4)	81 (18.3)	59 (20.6)	
England: Central/Midlands	102 (12.9)	53 (12.0)	22 (7.7)	
England: South	271 (34.4)	115 (26.0)	69 (24.1)	
Scotland	113 (14.3)	80 (18.1)	48 (16.8)	
Wales	96 (12.2)	83 (18.7)	68 (23.8)	
Northern Ireland	54 (6.8)	31 (7.0)	20 (7.0)	
Qualification				<0.001
Secondary education or less	336 (42.6)	237 (53.5)	178 (62.2)	
Further education	105 (13.3)	62 (14.0)	29 (10.1)	
Higher education	309 (39.2)	114 (25.7)	58 (20.3)	
Other	39 (4.9)	30 (6.8)	21 (7.3)	
Marital status				<0.001
Single, never married	81 (10.3)	34 (7.7)	23 (8.0)	
Married or partnership	495 (62.7)	258 (58.2)	134 (46.9)	
Divorced or widowed	213 (27.0)	151 (34.1)	129 (45.1)	
Smoking status (cigarettes)				<0.001
Never smoker	483 (61.2)	222 (50.1)	127 (44.4)	
Former smoker	251 (31.8)	143 (32.3)	91 (31.8)	
Current smoker	55 (7.0)	78 (17.6)	68 (23.8)	
Self-assessed general health				<0.001
Good	654 (82.9)	311 (70.2)	155 (54.2)	
Fair	122 (15.5)	102 (23.0)	102 (21.5)	
Bad	13 (1.7)	30 (6.8)	29 (10.1)	
Has longstanding illness, *yes*	354 (44.9)	248 (56.0)	193 (67.5)	<0.001
Number of medicines				<0.001
No medication	290 (36.8)	114 (25.7)	47 (16.4)	
1–4 medicines	389 (49.3)	206 (46.5)	113 (39.5)	
5 or more medicines	110 (13.9)	123 (27.8)	126 (44.1)	
Any dietary supplement use last year, *yes*	386 (48.9)	146 (33.0)	83 (29.0)	<0.001
Any of own teeth, *yes*	722 (91.5)	374 (84.4)	216 (75.5)	<0.001
Appetite				<0.001
Good	342 (43.4)	170 (38.4)	63 (22.0)	
Average	132 (16.7)	80 (18.1)	54 (18.9)	
Poor	7 (0.9)	18 (4.1)	24 (8.4)	
N/A to survey year	308 (39.0)	175 (39.5)	145 (50.7)	
BMI (kg/m^2), mean ± SD [2]	27.7 ± 4.6	28.7 ± 5.2	29.1 ± 5.9	<0.001
BMI (kg/m^2)				<0.001
≥20 (age < 70 years) or ≥22 (age ≥ 70 years)	739 (93.7)	398 (89.8)	238 (83.2)	
<20 (age < 70 years) or <22 (age ≥ 70 years)	23 (2.9)	12 (2.7)	18 (6.3)	
Unknown	27 (3.4)	33 (7.5)	30 (10.5)	
Protein intake (g)				<0.001
≥RNI	653 (82.8)	300 (67.7)	151 (51.8)	
<RNI	115 (14.6)	114 (25.7)	113 (39.5)	

Unknown	21 (2.7)	29 (6.6)	22 (7.7)	
Energy intake (kcal)				0.418
≥EAR	128 (16.2)	60 (13.5)	41 (14.3)	
<EAR	661 (83.8)	383 (86.5)	245 (85.7)	
Protein intake (g) and energy intake (kcal)				<0.001
≥RNI and ≥EAR	126 (16.0)	56 (12.6)	35 (12.2)	
<RNI and <EAR	115 (14.6)	113 (25.5)	110 (38.5)	
Either <RNI or <EAR	527 (66.8)	245 (55.3)	119 (41.6)	
Unknown	21 (2.7)	29 (6.6)	22 (7.7)	
Fruit and vegetable intake [3]				
<5 portions (80 g)/day	359 (45.5)	147 (33.2)	68 (23.8)	<0.001
<2 portions (80 g)/day	66 (8.4)	81 (18.3)	75 (26.2)	<0.001
Fluid intake [3]				
<1600 mL/day (women) and <2000 mL/day (men)	438 (55.5)	280 (63.2)	190 (66.4)	0.001
<1250 mL/day	174 (22.1)	113 (25.5)	74 (25.9)	0.257
<750 mL/day	21 (2.7)	14 (3.2)	9 (3.2)	0.849

Data are presented as number (%) or mean ± SD.

[1] Chi-square test for categorical variables and ANOVA test for continuous variables

[2] Based on 762 valid observations for no micronutrient deficiencies, 410 valid observations for one micronutrient deficiency and 256 valid observations for two micronutrient deficiencies.

[3] No unknown categories, remaining number (%) of subjects are ≥ the cut-off point

BMI Body Mass Index. RNI Recommended Nutrient Intake. EAR Estimated Average Requirement.

Supplementary Table S3. Comparison of study population characteristics between subjects included for analyses (n = 1518) and the total population (n = 3284), in order to explore generalizability of the current study results

Characteristic	Included for analysis (n = 1518)	Total population (n = 3284)
Sex, *women*	868 (57.2)	1878 (57.2)
Age group		
50–59 years	576 (37.9)	1171 (35.7)
60–69 years	522 (34.4)	1028 (31.3)
≥70 years	420 (27.7)	1085 (33.0)
Ethnic group, *white British*	1467 (96.6)	3156 (96.1)
Region		
England: North	293 (19.3)	587 (17.9)
England: Central/Midlands	177 (11.7)	382 (11.6)
England: South	455 (30.0)	910 (27.7)
Scotland	241 (15.9)	520 (15.8)
Wales	247 (16.3)	502 (15.3)
Northern Ireland	105 (6.9)	383 (11.7)
Qualification		
Secondary education or less	751 (49.5)	1846 (56.2)
Further education	196 (12.9)	353 (10.7)
Higher education	481 (31.7)	880 (26.8)
Other	90 (5.9)	205 (6.2)
Marital status		
Single, never married	138 (9.1)	339 (10.3)
Married or partnership	887 (58.4)	1788 (54.4)
Divorced or widowed	493 (32.5)	1157 (35.2)
Smoking status (cigarettes)		
Never smoker	832 (54.8)	1700 (51.8)
Former smoker	485 (32.0)	1085 (33.0)
Current smoker	201 (13.2)	499 (15.2)
Self-assessed general health [1]		
Good	1120 (73.8)	2213 (67.4)
Fair	326 (21.5)	811 (24.7)
Bad	72 (4.7)	249 (7.9)
Has longstanding illness, *yes*	795 (52.4)	1861 (56.7)
Number of medicines		
No medication	451 (29.7)	1464 (44.6)
1–4 medicines	708 (46.6)	1100 (33.5)
5 or more medicines	359 (23.7)	720 (21.9)
Any dietary supplement use last year, *yes*	615 (40.5)	1201 (36.6)
Any of own teeth, *yes*	1312 (86.4)	2752 (83.8)
Appetite [2]		
Good	575 (37.9)	1181 (36.0)
Average	266 (17.5)	613 (18.7)
Poor	49 (3.2)	134 (4.1)
N/A to survey year	628 (41.4)	1355 (41.3)
BMI (kg/m^2) [3]	28.2 ± 5.0	28.5 ± 5.2
BMI (kg/m^2)		
≥20 (age < 70 years) or ≥22 (age ≥ 70 years)	1375 (90.6)	2832 (85.4)
<20 (age < 70 years) or <22 (age ≥ 70 years)	53 (3.5)	132 (4.0)
Unknown	90 (5.9)	352 (10.6)
Protein intake (g)		
≥RNI	1104 (72.7)	2176 (66.3)
<RNI	342 (22.5)	820 (25.0)
Unknown	72 (4.7)	288 (8.8)

Energy intake (kcal)		
≥EAR	229 (15.1)	453 (13.8)
<EAR	1289 (84.9)	2831 (86.2)
Protein intake (g) and energy intake (kcal)		
≥RNI and ≥EAR	217 (14.3)	415 (12.6)
<RNI and <EAR	338 (22.3)	811 (24.7)
Either <RNI or <EAR	891 (58.7)	1770 (53.9)
Unknown	72 (4.7)	288 (8.8)
Fruit and vegetable intake [4]		
<5 portions (80 g)/day	944 (62.2)	2218 (67.5)
<2 portions (80 g)/day	222 (14.6)	626 (19.1)
Fluid intake [4]		
<1600 mL/day (women) and <2000 mL/day (men)	908 (59.8)	2062 (62.8)
<1250 mL/day	361 (23.8)	938 (28.6)
<750 mL/day	44 (2.9)	157 (4.8)
Haemoglobin (g/dL) [5]	13.8 ± 1.3	13.8 ± 1.3
Total Cholesterol (mmol/L) [6]	5.28 ± 1. 17	5.28 ± 1.18
CRP (mg/L) [7]	4.44 ± 6.63	4.55 ± 6.74
HbA1c (%) [8]	5.8 ± 0.8	5.8 ± 0.8
Ferritin (μg/L) [9]	118.1 ± 126.4	119.2 ± 127.9
25-Hydroxy Vitamin D (nmol/L) [10]	46.3 ± 21.9	46.2 ± 22.1

Data are presented as number (%) or mean ± SD.
[1] Frequencies of total population based on n = 3273
[2] Frequencies of total population based on n = 3283.
[3] Mean value based on n = 1428 and n = 2932
[4] No unknown categories, remaining number (%) of subjects are ≥ the cut-off point
[5] Mean value based on n = 1436 and n = 1663
[6] Mean value based on n = 1490 and n = 1709
[7] Mean value based on n = 1300 and n = 1497
[8] Mean value based on n = 1429 and n = 1656
[9] Mean value based on n = 1512 and n = 1699
[10] Mean value based on n = 1481 and n = 1654
BMI Body Mass Index. CRP C-Reactive Protein. HbA1c Haemoglobin A1C.

Reference

73 Vuilleumier, J.; Keck, E. Fluorometric assay of vitamin C in biological materials using a centrifugal analyser with fluorescence attachment. *J. Micronutr. Anal.* **1989**, 34, 599–626.
